# MicroRNA-30a-5p^me^: a novel diagnostic and prognostic biomarker for clear cell renal cell carcinoma in tissue and urine samples

**DOI:** 10.1186/s13046-020-01600-3

**Published:** 2020-06-01

**Authors:** Gonçalo Outeiro-Pinho, Daniela Barros-Silva, Elena Aznar, Ana-Isabel Sousa, Márcia Vieira-Coimbra, Jorge Oliveira, Céline S. Gonçalves, Bruno M. Costa, Kerstin Junker, Rui Henrique, Carmen Jerónimo

**Affiliations:** 1 Cancer Biology and Epigenetics Group, IPO Porto Research Center (CI-IPOP), Portuguese Oncology Institute of Porto (IPO Porto), Rua Dr. António Bernardino de Almeida, 4200-072 Porto, Portugal; 2 Master in Molecular Medicine and Oncology, Faculty of Medicine-University of Porto (FMUP), Porto, Portugal; 3 Instituto Interuniversitario de Investigación de Reconocimiento Molecular y Desarrollo Tecnológico (IDM), Universitat de València, CIBER de Bioingeniería, Biomateriales y Nanomedicina (CIBER-BBN), Camino de Vera s/n, 46022 Valencia, Spain; 4 Department of Urology, Portuguese Oncology Institute of Porto (IPO Porto), Rua Dr. António Bernardino de Almeida, 4200-072 Porto, Portugal; 5 Life and Health Sciences Research Institute (ICVS), School of Medicine, University of Minho, Campus de Gualtar, Braga, Portugal; 6 ICVS/3B’s - PT Government Associate Laboratory, Braga/Guimarães, University of Minho, Campus de Gualtar, Braga, Portugal; 7 Department of Urology and Pediatric Urology, Saarland University, Homburg, Saar Germany; 8 Department of Pathology, Portuguese Oncology Institute of Porto, Porto, Portugal; 9 Department of Pathology and Molecular Immunology, Institute of Biomedical Sciences Abel Salazar-University of Porto (ICBAS-UP), Rua de Jorge Viterbo Ferreira n.° 228, 4050-313 Porto, Portugal

**Keywords:** microRNA, DNA methylation, Clear cell renal cell carcinoma, Biomarker, Diagnosis, Prognosis

## Abstract

**Background:**

The rising incidence of renal cell carcinomas (RCC) constitutes a significant challenge owing to risk of overtreatment. Because aberrant microRNA (miR) promoter methylation contributes to cancer development, we investigated whether altered miR-30a-5p expression associates with DNA promoter methylation and evaluated the usefulness as clear cell RCC (ccRCC) diagnostic and prognostic markers.

**Methods:**

Genome-wide methylome and RNA sequencing data from a set of ccRCC and normal tissue samples from The Cancer Genome Atlas (TCGA) database were integrated to identify candidate CpG loci involved in cancer onset. MiR-30a-5p expression and promoter methylation were quantitatively assessed by PCR in a tissue set (Cohort #1) and urine sets (Cohorts #2 and 3) from IPOPorto and Homburg University Hospital. Non-parametric tests were used for comparing continuous variables. MiR-30a-5p promoter methylation (miR-30a-5p^me^) performance as diagnostic (receiver operator characteristics [ROC] - validity estimates) and prognostic [metastasis-free (MFS) and disease-specific survival (DSS)] biomarker was further validated in urine samples from ccRCC patients by Kaplan Meier curves (with log rank) and both univariable and multivariable analysis.

**Results:**

Two significant hypermethylated CpG loci in TCGA ccRCC samples, correlating with miR-30a-5p transcriptional downregulation, were disclosed. MiR-30a-5p^me^ in ccRCC tissues was confirmed in an independent patient’s cohort of IPOPorto and associated with shorter time to relapse. In urine samples, miR-30a-5p^me^ levels identified cancer both in testing and validation cohorts, with 83% sensitivity/53% specificity and 63% sensitivity/67% specificity, respectively. Moreover, higher miR-30a-5p^me^ levels independently predicted metastatic dissemination and survival.

**Conclusion:**

To the best of our knowledge, this is the first study validating the diagnostic and prognostic potential of miR-30a-5p^me^ for ccRCC in urine samples, providing new insights for its clinical usefulness as non-invasive cancer biomarker.

## Background

Renal cell carcinoma (RCC) is the third most prevalent urologic malignancy [1], twice more common in men than in women [2] disclosing rising incidence (2–4% per year) worldwide. RCC accounts for 2–3% of all malignant tumors in adults, displaying the highest mortality rate among urinary tract cancers [1, 3]. Notwithstanding, renal cell tumors are morphologically and genetically heterogeneous [4]. The three main subtypes of RCC - clear cell RCC (ccRCC), papillary RCC and chromophobe RCC [1] - have distinct clinical behaviors, which should be considered for adequate patient management [4].

CcRCC is simultaneously the most common and one of the most aggressive RCC subtypes, being prone to local invasion, metastization and death [2, 5]. It comprises 70–75% of all RCC cases and is characterized by several distinct genetic and epigenetic alterations [6]. About 25% of ccRCC patients present distant metastases at time of diagnosis, and in 20–50%, metastatic disease develops few years after diagnosis and surgical treatment of the primary tumor [3, 5, 7, 8]. Furthermore, ccRCC is extremely resistant to radiation and to conventional chemotherapy [3]. Therefore, biomarkers allowing for earlier diagnosis and accurate prognostication are required, improving current treatment and follow-up strategies [9]. Indeed, metastatic dissemination is the most important prognostic factor in ccRCC [10], highlighting the importance of accurately identifying patients at high risk of disease progression. Moreover, the identification of molecular biomarkers that might indicate risk of disease progression (recurrence, metastization) at the time of diagnosis might improve clinical management [3, 7] effectively contributing to implementation of Precision Medicine [10].

MicroRNAs (miRs) are small non-coding RNAs, 18–25 nucleotides long, that repress specific genes’ expression by targeting its 3′ untranslated region [11–13]. MiRs’ deregulation has been shown to participate in tumorigenesis, affecting differentiation, invasion, migration and apoptosis [10, 14] and has been implicated in urological tumors [15]. Several studies have associated microRNAs (miRs) deregulation with ccRCC clinicopathological features, suggesting a role in tumor initiation and progression [5, 7, 16, 17]. MiR-30a-5p, an intergenic miR (chromosome 6, 71,403,551–71,403,621 [− strand]), was suggested to play a role in cellular differentiation and development [18], but its precise role remains largely unknown [19, 20]. In ccRCC, an onco-suppressor function was proposed for miR-30a-5p, since its downregulation was associated with metastasis development [5, 9]. Moreover, miR-30a-5p was found to inhibit autophagy, by targeting *BECN1*, the gene encoding for beclin-1, a key protein for autophagosome formation [3]. In addition, miR-30a-5p was shown to decrease tumor microvessel density, by targeting endothelial DLL4, which is enrolled in tumor angiogenesis [5]. However, the mechanism underlying miR-30a-5p downregulation in ccRCC remains elusive [21]. Similarly to protein coding genes, miRs’ downregulation might be associated with aberrant promoter methylation, a common feature of urological tumors [22–25]. Thus, we sought to investigate for the first time, whether miR-30a-5p expression is regulated by promoter hypermethylation in ccRCC and evaluate its value as diagnostic and prognostic biomarker, both in tissue and urine samples.

## Materials and methods

### Patients and sample collection

Independent patient cohorts, two retrospective and one prospective, were selected for this study. Cohort #1 comprises 235 ccRCC patients, consecutively diagnosed and treated with nephrectomy, at Portuguese Oncology Institute of Porto (IPO Porto) between 2000 and 2017. For control purposes, normal kidney tissue from 25 patients subjected to nephrectomy due to upper urinary tract urothelial carcinoma was obtained. Tissue samples from primary tumors and normal kidney were collected immediately after surgery and promptly frozen at − 80 °C. Frozen tissue samples were cut in a cryostat and tumor cell content over 70% was confirmed in two hematoxylin and eosin stained slides taken before and after frozen section collection for nucleic acid extraction. A second cohort composed of 53 ccRCC patients, primarily diagnosed from 2007 to 2013 at IPO Porto, voluntarily provided 50 mL of voided urine samples (Cohort #2 - Testing). For control purposes, urine samples were collected from 57 healthy donors at IPO Porto (2009 to 2010). After collection, urine samples were centrifuged at 4000 rpm for 20 min at 4 °C and washed in PBS 1x. Lastly, pellets were frozen at − 80 °C. A third cohort (Cohort #3 – Validation) comprised 171 ccRCC patients, primarily diagnosed from 2015 to 2018 at Homburg University Hospital (Germany) provided, after informed consent, voided urine samples. For control purposes, urine samples were collected from 85 healthy donors at IPO Porto (2015–2017). After collection, 4 mL of whole urine was transferred into a tube and frozen at − 80 °C, until further usage.

Relevant clinical data was retrieved from clinical charts (Table 1). All procedures performed in studies involving human participants were performed in accordance with the ethical standards of the institutional ethics committee and with the 1964 Helsinki declaration and its later amendments or comparable ethical standards. Informed consent was obtained from all participants, according institutional regulations. This study was approved by the Institutional Review Board (Comissão de Ética para a Saúde) of IPO Porto, Portugal (CES-518/2010) and Jena University Hospital IRB.

**Table 1:** Clinicopathological data of tissue and urine samples used in this study

	Cohort #1 (Tissues)		Cohort #2 (Urines)		Cohort #3 (Urines)	
	ccRCC	RNT	ccRCC	AC	ccRCC	AC
Number of Patients, n	235	25	53	57	171	85
Median age, years (range)	65 (32–86)	71 (52–89)	61 (38–81)	49 (41–64	66 (36–87)	55 (45–65
ccRCC, n (%)						
ccRCCm	6 (2.55)	n.a.	15 (28.3)	n.a.	11 (6.4)	n.a.
Non-ccRCCm	229 (97.45)	n.a.	38 (71.6)	n.a.	160 (93.6)	n.a.
Stage, n (%)						
I	127 (54.0)	n.a.	26 (49.1)	n.a.	121 (70.8)	n.a.
II	33 (14.0)	n.a.	4 (7.5)	n.a.	8 (4.7)	n.a.
III	69 (29.4)	n.a.	18 (33.9)	n.a.	31 (18.1)	n.a.
IV	6 (2.6)	n.a.	5 (9.5)	n.a.	11 (6.4)	n.a.
Fuhrman Grade, n (%)						
1	7 (3.0)	n.a.	2 (3.8)	n.a.	8 (4.7)	n.a.
2	99 (42.1)	n.a.	26 (49.1)	n.a.	46 (26.9)	n.a.
3	104 (44.3)	n.a.	18 (33.9)	n.a.	6 (3.5)	n.a.
4	25 (10.6)	n.a.	7 (13.2)	n.a.	2 (1.2)	n.a.
k.a	n.a.	n.a.	n.a.	n.a.	109 (63.7)	n.a.
Follow up						
Median, months (range)	61 (0–194)	n.a.	58.00 (2.00–91.00)	n.a.	n.a	n.a.
Patients without remission (%)	2 (0.85)	n.a.	5 (9.4)	n.a.	n.a	n.a.
Recurrence (%)	43 (18.3)	n.a.	10 (18.9)	n.a.	n.a	n.a.
Death due to ccRCC	39 (16.6)	n.a.	10 (18.9)	n.a.	n.a	n.a.

*ccRCC* Clear Cell Renal Cell Carcinoma; *RNT* Renal Normal Tissue; *AC* Asymptomatic Control; *n.a* not applicable

### TCGA data analysis in ccRCC patients

Data on miR-30a-5p expression and methylation from ccRCC tumors and matched normal tissue samples was retrieved from The Cancer Genome Atlas (TCGA) database. MicroRNA-30a-5p expression data from samples hybridized by the University of North Carolina, Lineberger Comprehensive Cancer Center, using Illumina HiSeq 2000 Sequencing system, were downloaded from data matrix including 516 ccRCC samples (http://tcga-data.nci.nih.gov/tcga/tcgaDownload.jsp). DNA methylation data from miR-30a locus was evaluated using Illumina Infinium Human DNA Methylation 450 array and includes the methylation levels of 319 ccRCC samples. The provided value was pre-processed and normalized according to “level 3” specifications of TCGA (TCGA FPKM-UQ value; see http://cancergenome.nih.gov/ for details). This data is available for download through the NCI GDC data portal (https://portal.gdc.cancer.gov/).

### DNA extraction, bisulfite modification, pre-amplification and quantitative methylation-specific PCR

DNA was extracted from all clinical samples using phenol-chloroform method. Bisulfite modification was performed using EZ DNA Methylation-Gold™ Kit (Zymo Research, Orange, CA, USA), that integrates DNA denaturation and bisulfite conversion processes into one-step, according to recommended protocol. For urine samples from cohort #3, a pre-amplification step was performed prior to the quantitative methylation-specific PCR. SsoAdvanced™ PreAmp Supermix (Bio-Rad Laboratories Inc., Hercules, CA, USA) was used, following manufacturer recommendations. In brief, 8 μL of DNA template was added to 12 μL of nuclease-free water, 5 μL of Preamplification assay pool and, 25 μL of SsoAdvanced PreAmp Supermix (2x) and pre-amplified for 12 cycles.

Quantitative Methylation-specific PCR (qMSP) assays were carried out in triplicates using Xpert Fast SYBR (Grisp, Porto, Portugal), according to recommended protocol. Sequence-specific primers used in this study were designed to include the two CpGs tested in TCGA database and synthesized by Sigma Aldrich (Sigma-Aldrich, St. Louis, MO, USA) (Supplementary Table 2). Furthermore, the primer’s coverage sites within the methylated gene are available in Supplementary Table 3. For each sample, miR-30a-5p^me^ status was normalized to the endogenous control β-Actin.

### RNA extraction

Samples were suspended in TRIzol® reagent (Invitrogen, Carlsbad, CA, USA) and chloroform (Merk Milipore, Burlington, MA, USA) was added after cells were lysed. RNA concentrations and purity ratios were determined using a NanoDrop ND-1000 spectrophotometer (NanoDrop Technologies, Wilmington, DE, USA). RNA samples were stored at − 80 °C until further usage.

### MicroRNAs expression assay

Reverse transcription (RT) was performed using TaqMan MicroRNA Reverse Transcription Kit (Applied Biosystems, Foster City, CA, USA) according to manufacturer’s instructions. Quantitative Real-Time PCR (RT-qPCR) was performed in triplicates using TaqMan Small RNA Assays for miR-30a-5p (Assay ID 000417, Thermo Fisher Scientific, Waltham, MA, USA) and Xpert Fast Probe (Grisp, Porto, Portugal), according to recommended protocol. For each sample, miR expression was normalized to endogenous control RNU48 (Assay ID: 001006, Thermo Fisher Scientific, Waltham, MA, USA).

### Statistical analysis

Differences in methylation and expression levels and relationships between clinical variables were assessed using Kruskal-Wallis and Mann-Whitney U non-parametric tests for multiple groups (more than two) and pairwise comparisons, respectively. In multiple comparisons, Bonferroni’s correction was applied for pairwise comparisons, dividing the original *P*-value by the number of groups. *P*-values were considered statistically significant if inferior to 0.05 for comparisons between two groups.

For miR-30a-5p^me^, receiver operator characteristics (ROC) curves were constructed by plotting the true positive (sensitivity) against the false-positive (1-specificity) rate, and area under the curve (AUC) was calculated. Specificity, sensitivity, and accuracy were determined. For this, the empirical cut-off obtained by ROC curve analysis [sensitivity + (1-specificity)] was established. This cut-off value combines the maximum sensitivity and specificity, ensuring perfect categorization of the samples as positive and negative for methylation test.

Disease-free survival, disease-specific survival, and metastasis-free survival curves (Kaplan-Meier with log rank test) were constructed considering clinicopathological variables (stage and nuclear grade) and categorized miR-30a-5p^me^ or expression status. A Cox-regression model (multivariable model) was computed considering all significant clinical variables, to assess the relative contribution of each variable to the follow-up status.

Statistical analysis was performed using SPSS 25.0 for Windows (SPSS Inc., Chicago, IL, USA) and graphs were built using GraphPad Prism 6.0 software for Windows (GraphPad Software, San Diego, CA, USA).

## Results

### Analysis of miR-30a-5p^me^ and expression in ccRCC patients from the Cancer genome atlas

**Fig. 1:**
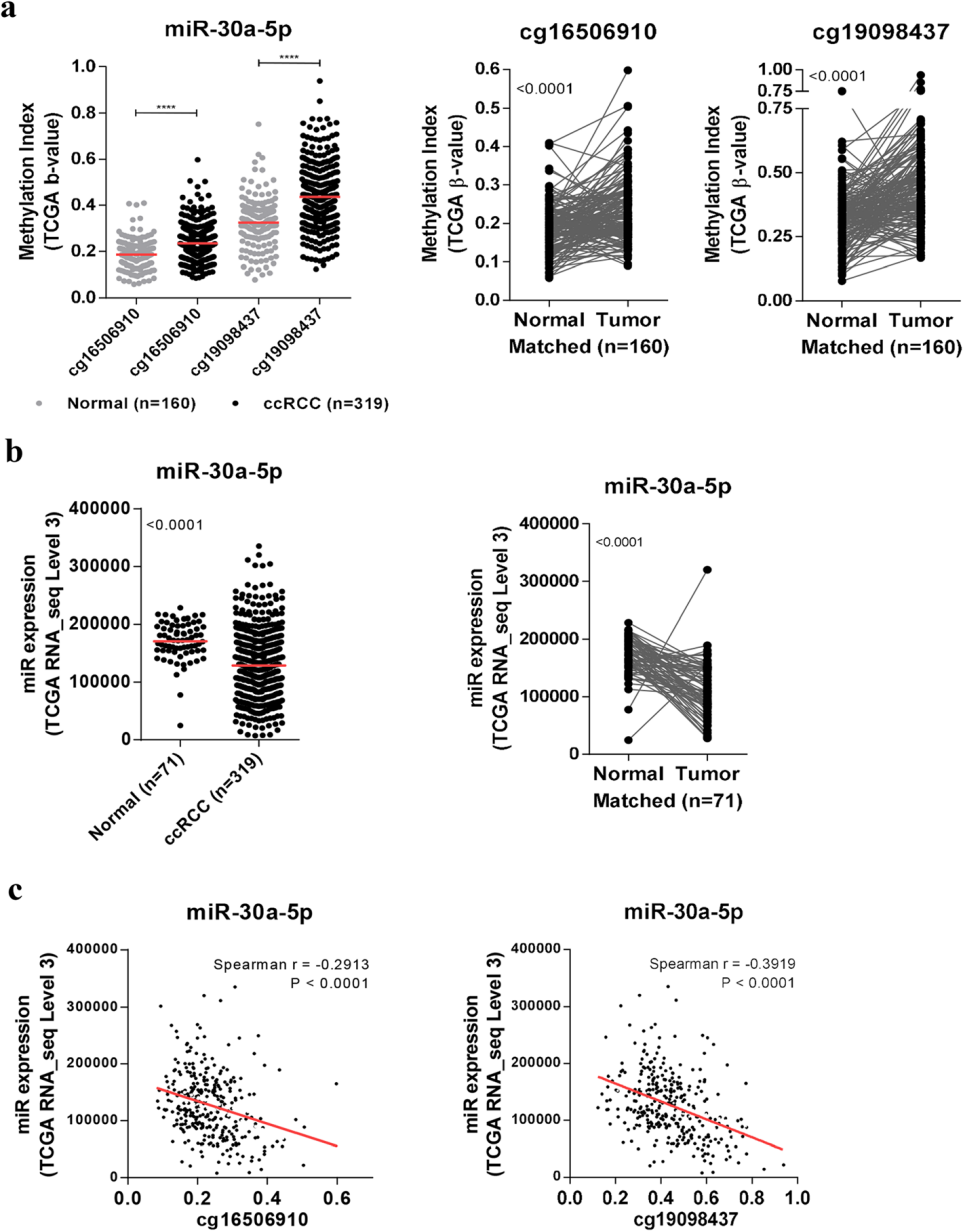
TCGA in silico analysis. **a** DNA methylation levels (β-Values) for each probe in specific miR loci, comparing normal and ccRCC samples (TCGA Illumina Infinium Human DNA Methylation 450 array), both in the whole cohort (*n* = 319 ccRCC) and in matched tumor/normal tissue (*n* = 160); **b** TCGA RNA-seq data for miR-30a-5p expression in ccRCC samples compared to normal samples, both patient-unmatched (*n* = 319 ccRCC) and -matched tumor/normal tissue (*n* = 71); **c** Correlation between miR-30a-5p expression and methylation levels for each probe using TCGA dataset for ccRCC tumor samples

The analysis of miR-30a^me^ locus in a cohort of ccRCC patients/samples from The Cancer Genome Atlas (TCGA) database identified two CpG sites with significantly higher methylation index (β-values) in tumor samples compared to normal controls [(*P* < 0.0001) (Fig. 1 (A)]. Conversely, miR-30a-5p expression levels were significantly downregulated in ccRCC when compared to non-cancerous tissue, both patient-unmatched and -matched [(*P* < 0·0001) (Fig. 1 (B)]. Furthermore, a significant inverse correlation between miR-30a-5p expression and promoter methylation was found [(*P* < 0.0001) (Fig. 1 (C)]. In univariable analysis, both miR-30a-5p expression and promoter methylation independently predicted worse overall survival (OS) and shorter recurrence-free survival (RFS). Similarly, both molecular variables retained their predictive value for OS, after adjusting for gender, age and stage, in multivariable analysis. Of note, miR-30a-5p^me^ also remained an independent predictor of shorter RFS in this model, as well as when tumor grade was included (Supplementary Table 1).

### MiR-30-5p expression and methylation status in tissues from ccRCC patients (cohort #1)

**Fig. 2:**
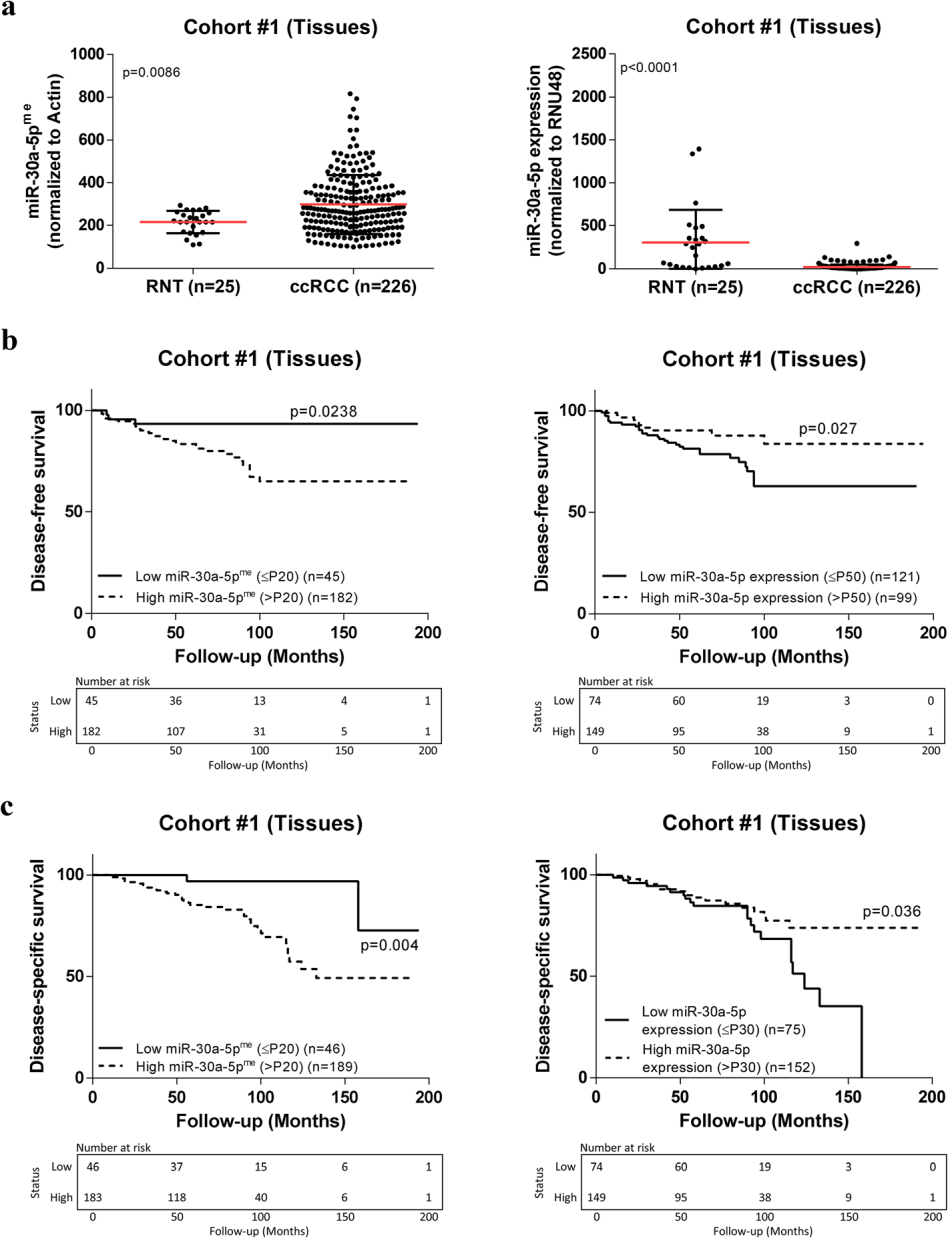
MiR-30a-5p promoter methylation levels, miR-30a-5p expression levels, and prognostic value in Cohort #1. **a** Scatter plots representing relative miR-30a-5p^me^ and expression levels between RNT (*n* = 25) and ccRCC (*n* = 226) samples (Mann–Whitney U test); **b** Disease-free and **c** disease-specific Kaplan-Meier survival curves based on miR-30a-5p^me^ and expression levels (Log-rank test)

Both promoter methylation and expression levels were assessed in an independent set of fresh-frozen tissue samples from IPO-Porto (Cohort #1). Corroborating the results from TCGA dataset, miR-30a-5p^me^ levels were significantly higher in ccRCC, compared to normal renal tissues (RNT) [(*P* = 0.0086) (Fig. 2 (A)], whereas miR-30a-5p expression levels were significantly lower in ccRCC samples [(*P* < 0.0001) (Fig. 2 (B)]. Nonetheless, no significant inverse correlation was found between miR’s promoter methylation and expression, in this IPO Porto cohort.

Furthermore, lower miR-30a-5p expression levels significantly associated with synchronous metastatic dissemination (*P* = 0.0447), recurrence (*P* = 0.0078) and higher nuclear grade (*P* = 0.0424) (Supplementary Figure 1). Conversely, no significant association was found between miR-30a-5p^me^ levels and classical clinical indexes, namely synchronous metastatic dissemination (*p* = 0.545), recurrence (*p* = 0.128), Führman Grade (*p* = 0.165), stage (*p* = 0.816), tumor size (*p* = 0.161) and age (*p* = 0.305).

### Assessment of miR-30a-5p^me^ and expression levels as prognostic marker for ccRCC

The prognostic value of miR-30a-5p expression and promoter methylation levels were also tested. The median follow-up time of IPO Porto Cohort #1 was 61 months, range (0–194) (Table 1). For disease-free survival (DFS) analysis, seven patients were excluded, five owing to the presence of metastasis at diagnosis and two because they never accomplished disease remission. In univariable analysis, advanced pathological stage and higher nuclear grade associated with DFS [*P* < 0.0001 and *P* = 0.015, respectively; Kaplan-Meier curves shown in Supplementary Figure 2 (A) and hazard ratio from COX regression shown in Supplementary Table 4] and disease-specific survival (DSS) [*P* < 0·0001 and *P* = 0.003, respectively; Kaplan-Meier curves shown in Supplementary Figure 2 (B) and hazard ratio from COX regression shown in Supplementary Table 4], as expected. Importantly, lower miR-30a-5p expression levels (below 50th percentile) and increased miR-30a-5p^me^ levels (over 20th percentile) significantly associated with shorter time to relapse [*P* = 0.030 and *P* = 0.035, respectively; (Fig. 2 (B)] and shorter DSS [*P* = 0.040 and *P* = 0.012, respectively; Fig. 2 (C)]. In multivariable analysis, only pathological stage, nuclear grade and miR-30a-5p^me^ levels (20th percentile) retained independent prognostic value for DSS (Table 2; Supplementary Table 4 and 5).

**Table 2:** Cox regression model assessing the prognostic potential of clinical and epigenetics variables in Cohort #1

Disease-Specific Survival	Variable	Hazard ratio (HR)	95% CI for OR	*P* value
**Multivariable**	**Stage** III/IV	3.048	1.586–5.858	0.001
	**Führman Grade** G3/G4	2.541	1.099–5.873	0.029
	**miR-30a-5p**^**me**^ ≥P20 vs. <P20	5.174	1.228–21.808	0.025

### MiR-30a-5p^me^ levels in urine sediments (cohort #2 – testing set)

**Fig. 3:**
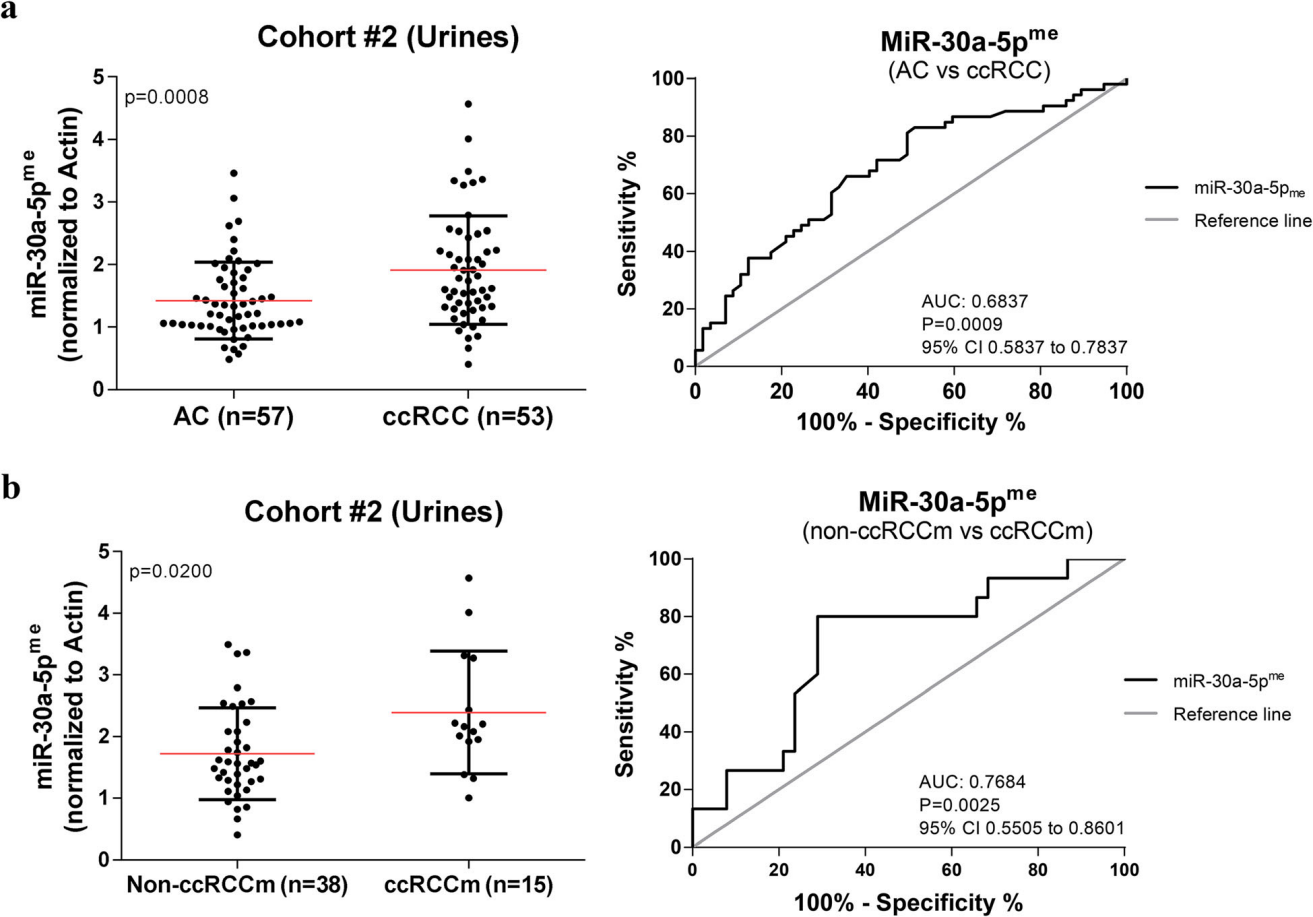
MiR-30a-5p^me^ levels and diagnostic value in Cohort #2 **a** Scatter plots (Mann–Whitney U test) and ROC curves for miR-30a-5p^me^ in AC (*n* = 57) and ccRCC (*n* = 53) and in **b** non-metastasized ccRCC (non-ccRCCm) (*n* = 38) and metastasized ccRCC (ccRCCm) (*n* = 15)

MiR-30a-5p^me^ was then tested in an independent set of urine sediment samples from a retrospective cohort of patients from IPO Porto (Cohort #2 - Testing). Significantly higher miR-30a-5p^me^ levels were found in urines from ccRCC patients compared to asymptomatic controls (AC) [(*P* = 0.0008) (Fig. 3 (A)]. Moreover, miR-30a-5p^me^ levels identified malignancy with 83% sensitivity and 53% specificity, providing an overall accuracy of 67% [AUC of 0.6837, (*P* = 0.0009) (Fig. 3 (A)] (Table 3).

**Table 3:** Diagnostic performance of miR-30a-5p promoter methylation-based biomarker in Cohort #2

Parameters	Cohort #2 (Urines) %	
	AC vs. ccRCC	Non-ccRCCm vs. ccRCCm
Sensitivity	83	80
Specificity	53	71
Accuracy	67	73

Importantly, patients that presented advanced pathological stage at diagnosis [(*P* = 0.0073) (Supplementary Figure 3(A)], patients who recurred (*P* = 0.048) and those that developed metastasis during follow up [(*P* = 0.0200) (Fig. 3 (B)] disclosed higher miR-30a-5p^me^ levels. However, no further associations were found between miR-30a-5p^me^ levels and other variables, namely age and tumor size (*P* = 0.294 and *P* = 0.224, respectively).

Remarkably, urine miR-30a-5p^me^ levels discriminated patients with metastasis (both synchronous and metachronous) from those without metastatic disease with 80% sensitivity, 71% specificity and 73% accuracy [AUC of 0.7684, (*P* = 0.0025) (Fig. 3 (B)] (Table 3).

**Fig. 4:**
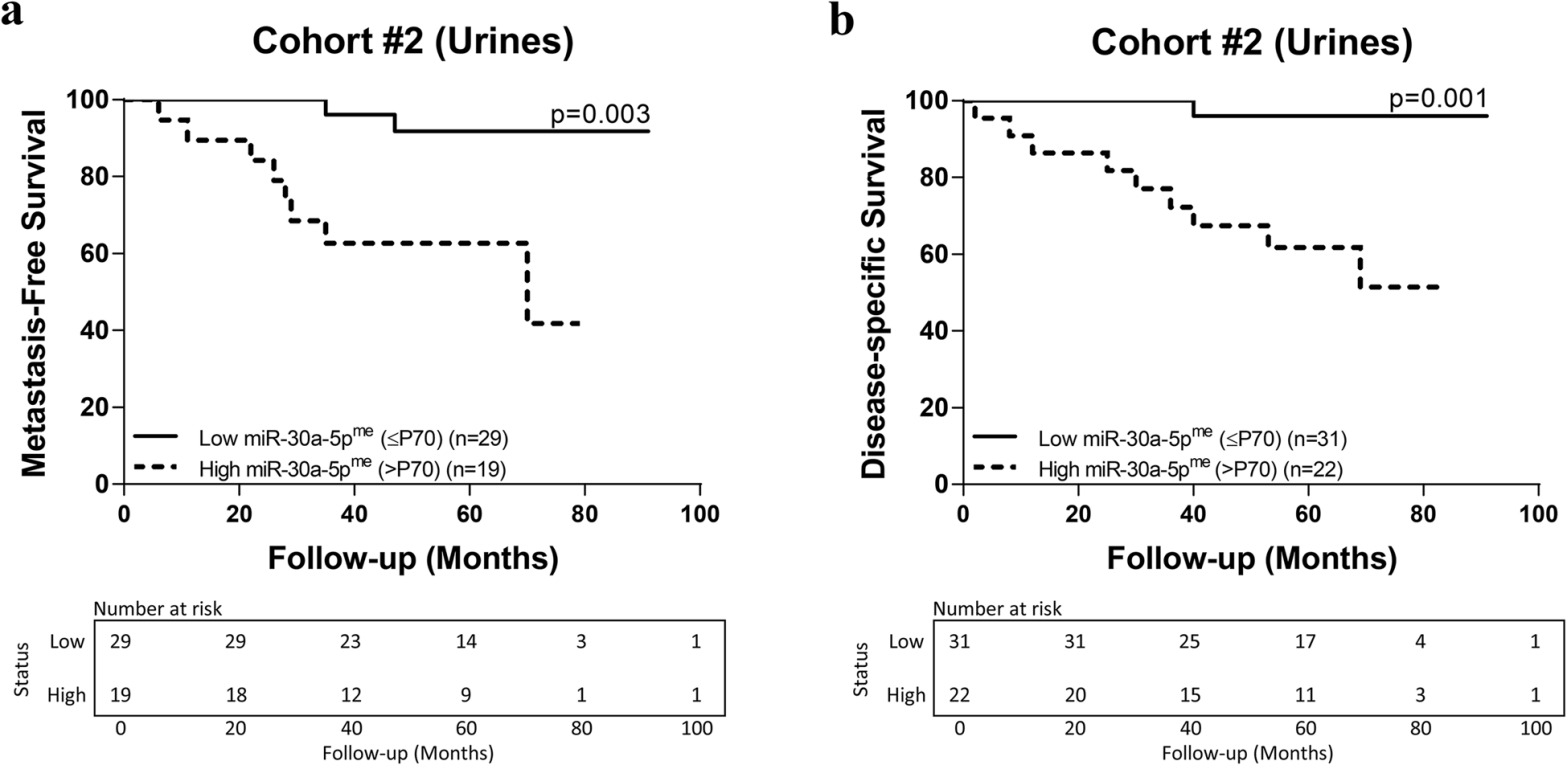
MiR-30a-5p^me^ prognostic value in Cohort #2. Metastasis-free and disease-specific Kaplan-Meier survival curves based on miR-30a-5p^me^ levels (Log-rank test)

The median follow-up for cohort #2 was 58 months, range (2–91) (Table 1). Five patients with metastatic disease at diagnosis were excluded for metastasis-free survival analysis. Worse DSS and shorter metastasis-free survival (MFS) was observed in patients with advanced pathological stage and higher nuclear grade [*P* < 0.0001 and *P* = 0.002, respectively; Supplementary Figure 3 (B)] and [*P* < 0.0001 and *P* = 0.032, respectively; Supplementary Figure 3 (C)]. Remarkably, higher miR-30a-5p^me^ (above 70th percentile) also associated with shorter MFS and worse DSS [*P* = 0.003 and *P* = 0.001, respectively; Fig. 4 (A) and (B)].

In univariable Cox-regression analysis, higher miR-30a-5p^me^levels (70th percentile) and nuclear grade predicted shorter MFS and DSS (Supplementary Tables 6 and 7). However, in multivariable analysis, these parameters only depicted independent prognostic value for DSS (Table 4).

**Table 4:** Cox regression model assessing the prognostic potential of clinical and epigenetics variables in Cohort #2

Disease-Specific Survival	Variable	HR	95% CI for HR	*p* value
**Multivariable**	**miR-30a-5p**^**me**^ ≥P70 vs. <P70	10.405	1.296–83.509	0.028
	**Führman Grade** G1&G2 vs. G3&G4	9.376	1.158–75.903	0.036

### MiR-30a-5p^me^ levels in urine supernatants (cohort #3 – validation set)

**Fig. 5:**
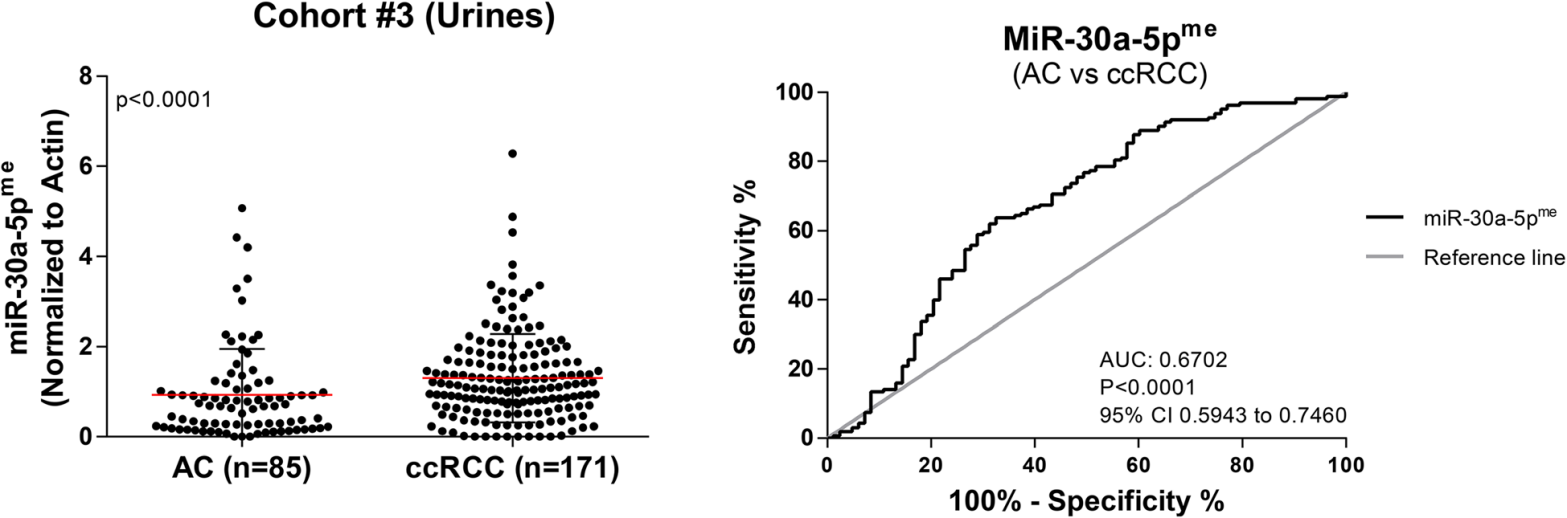
MiR-30a-5p^me^ levels and diagnostic value in Cohort #3. Scatter plots (Mann–Whitney U test) and ROC curves for miR-30a-5p^me^ in AC (*n* = 85) and ccRCC (*n* = 171)

Considering the results from Cohort #2 - Testing, we further assessed the detection performance of miR-30a-5p^me^ levels in a larger, independent set of urine from a prospective cohort of 171 ccRCC patients and 85 AC. Paralleling the previous observations, miR-30a-5p^me^ levels were significantly higher in ccRCC patients, comparing to AC (*P* < 0.0001) (Fig. 5). Furthermore, miR-30a-5p^me^ levels identified ccRCC with 63% sensitivity, 67% specificity and 63% accuracy [AUC of 0.6702, (*P* < 0.0001) (Fig. 5)] (Table 5). Despite miR-30a-5p^me^ levels were lower in ccRCC stages I & II comparatively to stages III & IV, the difference did not reach statistical significance. Moreover, miR-30a-5p^me^ did not significantly associate with either age or tumor size (*p* = 0.280 and *p* = 0.460, respectively.

**Table 5:** Diagnostic performance of miR-30a-5p promoter methylation-based biomarker in Cohort #3

Parameters	Cohort #3 (Urines) %
	AC vs. ccRCC
Sensitivity	63
Specificity	67
Accuracy	63

## Discussion

Over the last decade, the frequency of incidentally detected RCC has significantly increased, mostly due to the widespread use of imaging techniques. CcRCC, the most prevalent RCC, carries worse prognosis than other common RCC subtypes, as approximately 20–40% of cases develop distant metastases [7], which are the main cause of RCC-related mortality, the highest among urologic cancers [26, 27]. Thus, biomarkers capable of accurately identifying ccRCC cases prone to metastasize, mostly among early stage tumors at diagnosis, would be a major clinical breakthrough. MiR-30a-5p expression downregulation has been reported in ccRCC, associated with metastatic disease and adverse prognosis. This being said, we aimed to determine whether (a) miR-30a-5p expression silencing was due to aberrant promoter methylation and (b) miR-30a-5p^me^ levels might not only accurately detect ccRCC in tissue and urine samples, but also identify patients at increased risk to develop metastatic disease independently of standard clinicopathological parameters.

Firstly, TCGA dataset was surveyed and two CpG loci at miR-30a promoter were identified as putative regulators of its expression in ccRCC. Further, miR-30a-5p^me^ inversely correlated with miR-30a-5p expression levels in ccRCC. Moreover, these results were mostly corroborated in IPO Porto ccRCC cohort #1, also confirming previous reports [28, 29], and providing compelling evidence that miR-30a-5p downregulation in ccRCC might be caused by aberrant promoter methylation. Thus, our results add miR-30a-5p to the growing list of epigenetically-deregulated microRNAs in urologic malignancies [22–25], reinforcing the contribution of epigenetic alterations to renal carcinogenesis.

Notwithstanding the mechanism underlying miR-30a-5p downregulation in ccRCC, our study firstly demonstrated that miR-30a-5p^me^ levels might be a specific biomarker for this cancer type. Indeed, since high methylation levels are cancer-specific, they may be used as a tool for ccRCC identification, both in tissue (e.g., as an ancillary tool for histopathological or cytopathological workup of renal mass) and urine samples, providing, in the latter case, a non-invasive tool for early disease detection in high-risk populations [30, 31] (e.g., patients with end-stage chronic renal disease undergoing haemodialysis). Although other hypermethylated miRs (miR-9, miR-124-3) have been proposed as molecular biomarkers for ccRCC [32, 33], their performance in urine samples has not been assessed, yet. Thus, to the best of our knowledge, this is the first miR methylation-based urine biomarker to be proposed for ccRCC. Importantly, it should be emphasized that miR^me^ assessment has several advantages, including higher stability, reduced amount of clinical material requirements and methodological celerity compared to RNA expression assays. Thus, methylation analysis is more robust, enabling the development of tests for use in clinical practice [34, 35].

The rising incidence of incidentally detected RCC presents a significant clinical challenge owing to the risk of overtreatment. Thus, perfecting prognostic models through the inclusion of molecular biomarkers might contribute to reduce that risk. Remarkably, we demonstrated that higher miR-30a-5p^me^ levels assessed in tissue samples independently predicted shorter time to relapse, showing promise as biomarker for risk-stratification among ccRCC and more accurate identification of the high-risk patient subset which may require alternative therapeutic interventions.

Although miR-30a-5p^me^ biomarker performance in Cohort #3 was not impressive, it should be highlighted that miR-30a-5p^me^ levels were able to identify six out of each ten ccRCC in urine samples and, notably, correctly classified seven out of each ten suspects. This simple and cost-effective method is likely to increase patient compliance while reducing the risk of mistreatment. Moreover, since metastatic patients disclosed significantly higher miR-30a-5p^me^ levels than non-metastatic ccRCC, this non-invasive test might also provide relevant information concerning patient monitoring after curative-intent surgery.

The main limitation of our study is the relatively small number of urine samples tested. Moreover, accuracy might be improved by adding additional markers to the panel, as we previously demonstrated for other urologic cancers [22–25]. Nonetheless, the novelty of using a miRNA methylation marker with diagnostic and prognostic value, amenable for non-invasive detection, constitutes, in our view, a relevant contribution to the field and, hopefully, will stimulate the design of validation studies in larger and independent series.

## Conclusions

We demonstrated that miR-30a-5p downregulation, probably due to aberrant promoter methylation, is common in ccRCC. Importantly, miR-30a-5p^me^ levels might aid for diagnostic and prognostic purposes, helping to identify patients at higher risk for disease progression and metastization, although additional markers should be included to improve its overall performance as diagnostic/prognostic biomarker.

## Supplementary information

**Additional file 1 Supplementary Figure S1. Expression of miR-30a-5p according to clinicopathological variables in Cohort #1.** Scatter plots of miR-30a-5p expression levels according to metastasis presentation, recurrence and Führman grade (Mann–Whitney U test).

**Additional file 2 Supplementary Figure S2. Prognostic value of stage and nuclear grade in Cohort #1. (A)** Disease-free and (**B**) disease-specific Kaplan-Meier survival curves based on clinicopathological stage and nuclear grade (Log-rank test).

**Additional file 3 Supplementary Figure S3. MiR-30a-5p**^**me**^**levels and prognostic value of stage and nuclear grade in Cohort #2 (A)** Scatter plots of miR-30a-5p^me^ levels according to pathological stage (Mann–Whitney U test); **(B)** Disease-specific and **(C)** Metastasis-free Kaplan-Meier survival curves based on clinicopathological stage and nuclear grade (Log-rank test).

**Additional file 4 Supplementary Table 1.** Univariable and multivariable analysis of clinicopathological and epigenetic variables in TCGA Cohort (OS and RFS). **Supplementary Table 2.** List of primers’ sequence for qMSP analysis. **Supplementary Table 3.** qMSP primers’ design within the methylated gene’s sequence. **Supplementary Table 4.** Cox univariable and multivariable analysis of clinicopathological and epigenetic variables in Cohort #1 (DFS). **Supplementary Table 5.** Cox univariable analysis of clinicopathological and epigenetic variables in Cohort #1 (DSS). **Supplementary Table 6.** Cox univariable and multivariable analysis of clinicopathological and epigenetic variables in Cohort #2 (MFS). **Supplementary Table 7.** Cox univariable analysis of clinicopathological and epigenetic variables in Cohort #2 (DSS).

## Data Availability

The datasets analysed during the current study are available in the TCGA repository (http://tcga-data.nci.nih.gov/tcga/tcgaDownload.jsp).

## Abbreviations

AC: Asymptomatic controls
AUC: Area under the curve
ccRCC: Clear cell Renal Cell Carcinoma
DFS: Disease-free survival
DSS: Disease-specific survival
MFS: Metastasis-free survival
miR: MicroRNA
miR-30a-5p^me^: MiR-30a-5p promoter methylation
OS: Overall survival
qMSP: Quantitative methylation-specific PCR
RCC: Renal cell carcinoma
RT: Reverse transcription
RFS: Recurrence-free survival
RNT: Renal normal tissue
ROC: Receiver operator characteristics
TCGA: The Cancer Genome Atlas
