# Correction: MicroRNA-30a-5p^me^: a novel diagnostic and prognostic biomarker for clear cell renal cell carcinoma in tissue and urine samples

**DOI:** 10.1186/s13046-022-02450-x

**Published:** 2022-08-15

**Authors:** Gonçalo Outeiro-Pinho, Daniela Barros-Silva, Elena Aznar, Ana-Isabel Sousa, Márcia Vieira-Coimbra, Jorge Oliveira, Céline S. Gonçalves, Bruno M. Costa, Kerstin Junker, Rui Henrique, Carmen Jerónimo

**Affiliations:** 1 Cancer Biology and Epigenetics Group, IPO Porto Research Center (CI-IPOP), Portuguese Oncology Institute of Porto (IPO Porto), Rua Dr. António Bernardino de Almeida, 4200-072 Porto, Portugal; 2 Master in Molecular Medicine and Oncology, Faculty of Medicine-University of Porto (FMUP), Porto, Portugal; 3 Instituto Interuniversitario de Investigación de Reconocimiento Molecular y Desarrollo Tecnológico (IDM), Universitat de València, CIBER de Bioingeniería, Biomateriales y Nanomedicina (CIBER-BBN), Camino de Vera s/n, 46022 Valencia, Spain; 4 Department of Urology, Portuguese Oncology Institute of Porto (IPO Porto), Rua Dr. António, Bernardino de Almeida, 4200-072 Porto, Portugal; 5 Life and Health Sciences Research Institute (ICVS), School of Medicine, University of Minho, Campus de Gualtar, Braga, Portugal; 6 ICVS/3B’s - PT Government Associate Laboratory, Braga/Guimarães, University of Minho, Campus de Gualtar, Braga, Portugal; 7 Department of Urology and Pediatric Urology, Saarland University, Homburg, Saar Germany; 8 Department of Pathology, Portuguese Oncology Institute of Porto, Porto, Portugal; 9 Department of Pathology and Molecular Immunology, Institute of Biomedical Sciences Abel Salazar-University of Porto (ICBAS-UP), Rua de Jorge Viterbo Ferreira n. 228, 4050-313 Porto, Portugal

**Correction: J Exp Clin Cancer Res 39, 98 (2020)**

**https://doi.org/10.1186/s13046-020-01600-3**

Following publication of the original article [1], an error was identified in Supplementary Figure 1 where incorrectly labelled graphs were found; specifically:

- Supplementary Figure 1: the y axis is currently labelled as miR-30c-5p expression (normalized to RNU48) whereas it should be labelled as miR-30a-5p expression (normalized to RNU48); correct supplementary file is now used

The correction does not have any effect on the results or conclusions of the paper. The original article has been corrected.

## Supplementary Information

**Additional file 1: Supplementary Figure S1.** Expression of miR-30a-5p according to clinicopathological variables in Cohort #1. Scatter plots of miR-30a-5p expression levels according to metastasis presentation, recurrence and Führman grade (Mann–Whitney U test).

## References

[1] Outeiro-Pinho G, Barros-Silva D, Aznar E (2020). MicroRNA-30a-5p^me^: a novel diagnostic and prognostic biomarker for clear cell renal cell carcinoma in tissue and urine samples. J Exp Clin Cancer Res.

