# Supplementary figures and images for: MicroRNA-30a-5pme: a novel diagnostic and prognostic biomarker for clear cell renal cell carcinoma in tissue and urine samples
Source: J Exp Clin Cancer Res. 2020 Jun 1;39:98. doi: 10.1186/s13046-020-01600-3 (PMC7323611; doi:10.1186/s13046-020-01600-3)

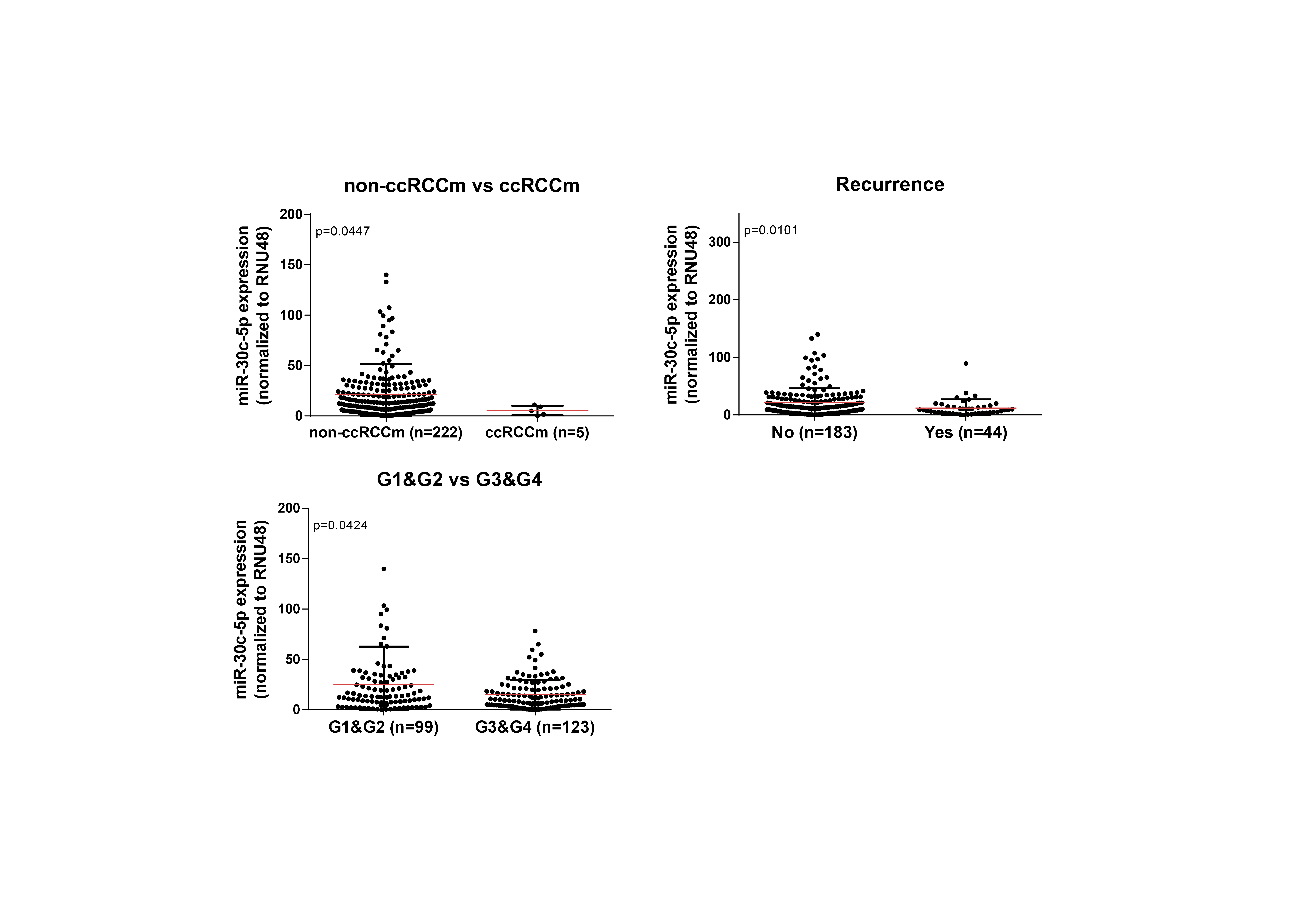

Supplement: Supplementary file 1 — Additional file 1 Supplementary Figure S1. Expression of miR-30a-5p according to clinicopathological variables in Cohort #1. Scatter plots of miR-30a-5p expression levels according to metastasis presentation, recurrence and Führman grade (Mann–Whitney U test). [file 13046_2020_1600_MOESM1_ESM.tif]

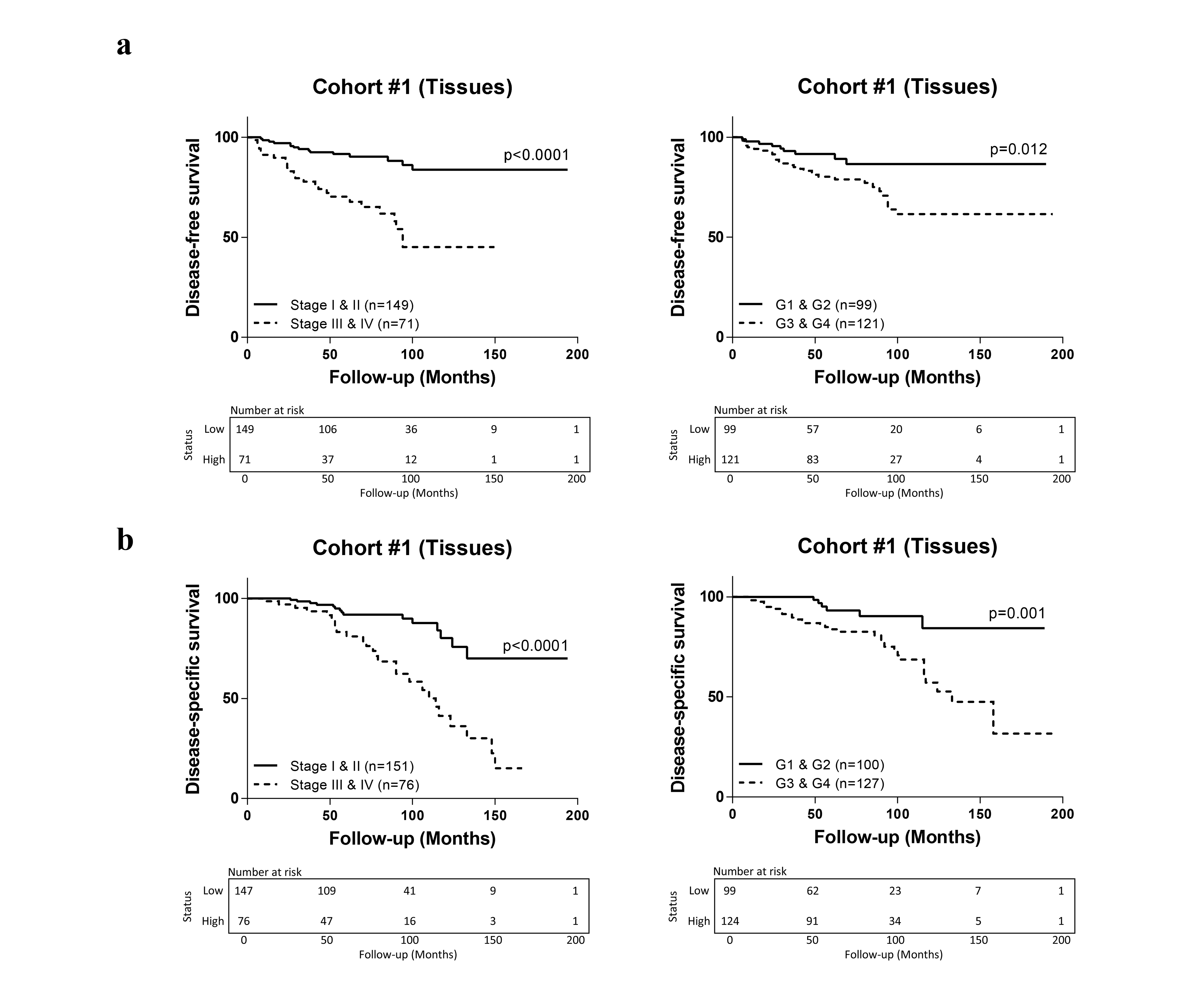

Supplement: Supplementary file 2 — Additional file 2 Supplementary Figure S2. Prognostic value of stage and nuclear grade in Cohort #1. (A) Disease-free and (B) disease-specific Kaplan-Meier survival curves based on clinicopathological stage and nuclear grade (Log-rank test). [file 13046_2020_1600_MOESM2_ESM.tif]

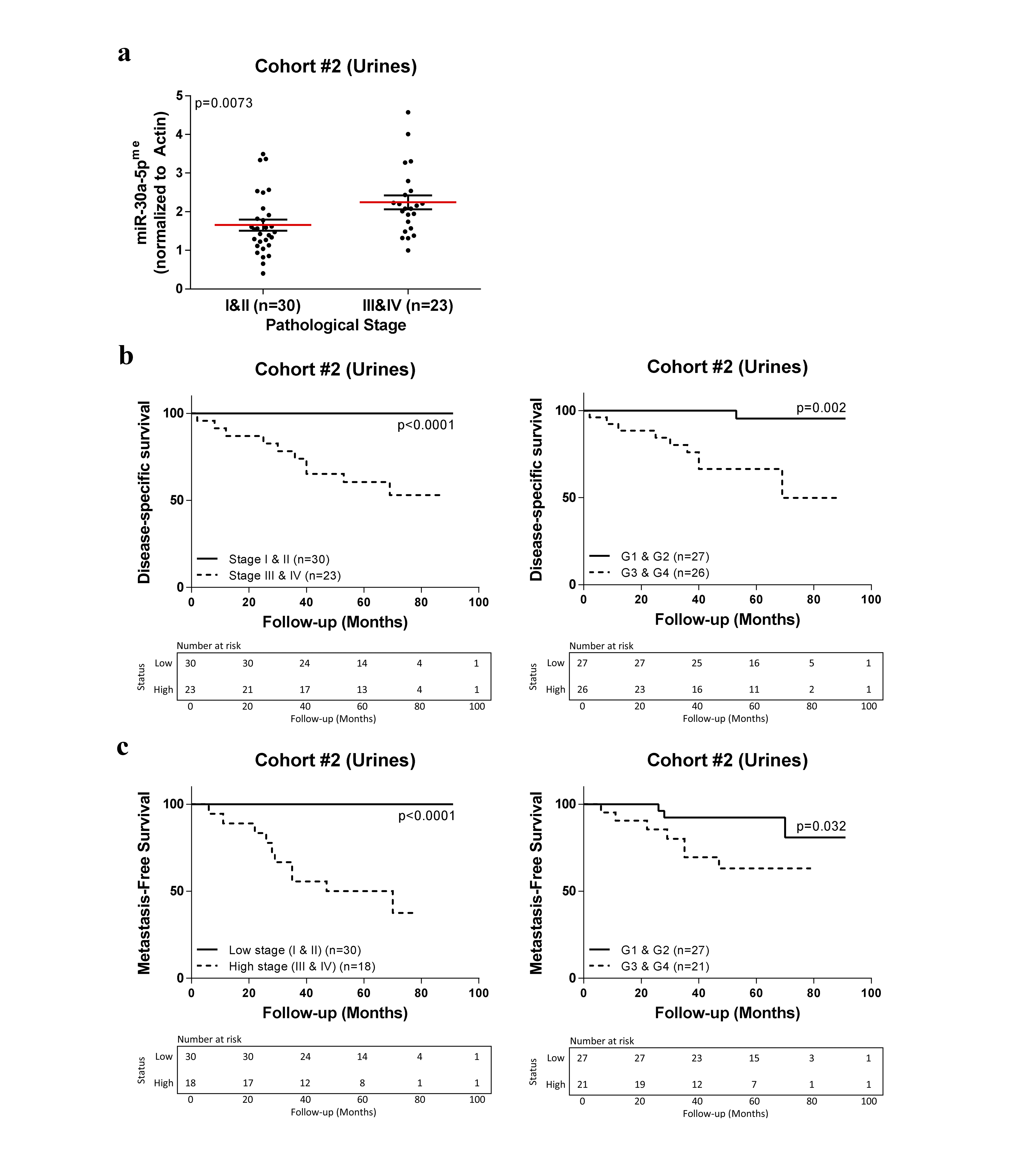

Supplement: Supplementary file 3 — Additional file 3 Supplementary Figure S3. MiR-30a-5pmelevels and prognostic value of stage and nuclear grade in Cohort #2 (A) Scatter plots of miR-30a-5pme levels according to pathological stage (Mann–Whitney U test); (B) Disease-specific and (C) Metastasis-free Kaplan-Meier survival curves based on clinicopathological stage and nuclear grade (Log-rank test). [file 13046_2020_1600_MOESM3_ESM.tif]
