# Supplementary material for: MicroRNA-30a-5pme: a novel diagnostic and prognostic biomarker for clear cell renal cell carcinoma in tissue and urine samples
Source: J Exp Clin Cancer Res. 2020 Jun 1;39:98. doi: 10.1186/s13046-020-01600-3 (PMC7323611; doi:10.1186/s13046-020-01600-3)
Supplement: Supplementary file 4 — Additional file 4 Supplementary Table 1. Univariable and multivariable analysis of clinicopathological and epigenetic variables in TCGA Cohort (OS and RFS). Supplementary Table 2. List of primers’ sequence for qMSP analysis. Supplementary Table 3. qMSP primers’ design within the methylated gene’s sequence. Supplementary Table 4. Cox univariable and multivariable analysis of clinicopathological and epigenetic variables in Cohort #1 (DFS). Supplementary Table 5. Cox univariable analysis of clinicopathological and epigenetic variables in Cohort #1 (DSS). Supplementary Table 6. Cox univariable and multivariable analysis of clinicopathological and epigenetic variables in Cohort #2 (MFS). Supplementary Table 7. Cox univariable analysis of clinicopathological and epigenetic variables in Cohort #2 (DSS). [file 13046_2020_1600_MOESM4_ESM.docx]

**Supplementary Materials:**

**Supplementary Table 1.** Univariable and multivariable analysis of clinicopathological and epigenetic variables in TCGA Cohort (OS and RFS).

|  |  |  | **OS** | | | | **RFS** | | | |  |
| --- | --- | --- | --- | --- | --- | --- | --- | --- | --- | --- | --- |
|  |  |  | **Univariable (log-rank)** | | **Multivariable (COX)** | | **Univariable (log-rank)** | | **Multivariable (COX)** | |  |
|  |  |  | **P** | **Prognosis** | **P** | **Exp(B)** | **P** | **Prognosis** | **P** | **Exp(B)** |  |
| **Expression** | **miR-30a-5p** | P10 | 0·004 | + | n.s. | n.s. | n.s. | n.s. | n.s. | n.s. | **These COX included gender, age (continuous), pTstage and grade** |
|  |  | P20 | 0.004 | + | n.s. | n.s. | 0.070 | + | n.s. | n.s. |  |
|  |  | P25 | 0.025 | + | n.s. | n.s. | n.s. | n.s. | n.s. | n.s. |  |
|  |  | P30 | 0.032 | + | n.s. | n.s. | 0.059 | + | n.s. | n.s. |  |
|  |  | P40 | n.s. | n.s. | n.s. | n.s. | 0.034 | + | n.s. | n.s. |  |
|  |  | P75 | n.s. | n.s. | n.s. | n.s. | 0.049 | + | n.s. | n.s. |  |
| **Methylation** | **cg16506910** | P50 | 0.011 | - | n.s. | n.s. | n.s. | n.s. | n.s. | n.s. |  |
|  |  | P60 | 0.010 | - | n.s. | n.s. | n.s. | n.s. | n.s. | n.s. |  |
|  |  | P70 | 0.005 | - | n.s. | n.s. | n.s. | n.s. | n.s. | n.s. |  |
|  |  | P75 | 0.014 | - | n.s. | n.s. | n.s. | n.s. | n.s. | n.s. |  |
|  |  | P80 | 0.009 | - | n.s. | n.s. | n.s. | n.s. | n.s. | n.s. |  |
|  |  | P90 | 0.001 | - | n.s. | n.s. | n.s. | n.s. | n.s. | n.s. |  |
|  | **cg19098437** | P10 | n.s. | n.s. | 0.082 | 0.520 | n.s. | n.s. | 0.064 | 0.466 |  |
|  |  | P20 | n.s. | n.s. | n.s. | n.s. | n.s. | n.s. | 0.005 | 0.418 |  |
|  |  | P25 | n.s. | n.s. | n.s. | n.s. | n.s. | n.s. | 0.017 | 0.480 |  |
|  |  | P30 | n.s. | n.s. | n.s. | n.s. | n.s. | n.s. | 0.011 | 0.466 |  |
|  |  | P40 | 0.006 | - | n.s. | n.s. | n.s. | n.s. | 0.001 | 0.398 |  |
|  |  | P50 | 0.001 | - | 0.069 | 1.487 | n.s. | n.s. | n.s. | n.s. |  |
|  |  | P60 | 0.000 | - | n.s. | n.s. | 0.086 | - | n.s. | n.s. |  |
|  |  | P70 | 0.005 | - | n.s. | n.s. | n.s. | n.s. | n.s. | n.s. |  |
|  |  | P75 | 0.026 | - | n.s. | n.s. | n.s. | n.s. | n.s. | n.s. |  |
|  |  | P80 | 0.002 | - | n.s. | n.s. | n.s. | n.s. | n.s. | n.s. |  |
|  |  | P90 | 0.000 | - | n.s. | n.s. | n.s. | n.s. | n.s. | n.s. |  |
| **Expression** | **miR-30a-5p** | P20 |  | | 0.046 | 0.694 |  | | n.s. | n.s. | **These COX included gender, age (continuous) and pTstage** |
| **Methylation** | **cg16506910** | P50 |  |  | 0.037 | 1.549 |  |  | n.s. | n.s. |  |
|  | **cg19098437** | P20 |  |  | n.s. | n.s. |  |  | 0.023 | 0.505 |  |
|  |  | P40 |  |  | 0.014 | 1.740 |  |  | 0.038 | 0.577 |  |
|  |  | P50 |  |  | 0.012 | 1.728 |  |  | n.s. | n.s. |  |
|  |  | P60 |  |  | 0.024 | 1.610 |  |  | n.s. | n.s. |  |
|  |  | P90 |  |  | 0.038 | 1.864 |  |  | n.s. | n.s. |  |

The “+” sign represents “good prognosis” whereas the “-“sign represents “bad prognosis”; n.s. – not significant

**Supplementary Table 2.** List of primers’ sequence for qMSP analysis.

| **Gene** | **Primer sequence** |
| --- | --- |
| **miR-30a-5p Forward**  **miR-30a-5p Reverse**  **β-actin Forward**  **β-actin Reverse** | 5’-TAGTCGAGGATGTTTATAG-3’  5’-AACTTCAATACTTTACAAAATCG-3’  5’-TGGTGATGGAGGAGGTTTAGTAAGT-3’  5’-ACCAATAAAACCTACTCCTCCCTTAA-3’ |

**Supplementary Table 3.** qMSP primers’ design within the methylated gene’s sequence.

| **Gene’s sequence** |
| --- |
| **T**TGTGG**T**TT**T**A**T**AG**T**TT**TT**AGTGAGGATGTTTA**T**AGT**C**G**T**T**T**A**T**TGT**T**AA**T**AG**T**AATATA**TT**TT**T**TTTAG**TT**TT**T**TGTTGGGTTAA**TT**TGAAGAAGTAAT**TTT**AG**T**AAGTGTTT**TT**AAGATGTG**T**AGG**T**AA**C**GATT**T**TGTAAAGTA**T**TGAAG**TT**T**T**ATT**T**AAA**T**AG**T**AATATT**T**TAGAAAATGT**T**T**T**AAATT**T**AAAA**T**AAATAAGAAGG**T**T**T**ATGGATGT**T**TATTATT**T**AT**T**A**TC**GAG**T**TT**T**TAGTTT**T**TATAAAATTTTGTGATATA**T**TAAAA**T**AA**T**ATATATAAATGGTTT**T**ATT**T**ATTATTGTTAG**TT**A**T**AAGAAGTAAATAGG**T**AAGTGG**TT**AGGTTA**T**TTAATATGATTT**T**AA**T**ATG**T**ATTATGTAATTA**T**AAT**TT**TTTAAG**T**AAAAGTGA**TT**AAA**T**A**T**AGAAA**T**TAAATTAT**T**TTAA**T**AG**C**GTAAAG**C**GTA**T**ATTTAA**T**AT**T**AAATA**T**TG**T**AGTTGAGTGAA**T**TTAGA**T**TA**TC**GTGAAAATATAAAAGGT**T**TA**T**TTGGAAAGTTATAAA**T**T**T**TGA**T**AAAAAAAAAAAAGA**T**TTA**T**ATG**T**TG**T**TTTTATAGAT**T**A**T**TTTTG**T**T**T**TAAAGT**T**TG**T**T**TTT**AGAGAGGA**T**TTGTA**T**AGT**T**AG**T**TGATTTTTGAAAGAGTAAG**T**AA**T**AAT**T**AGGAGAAAAA**T**AGAAAAGAATAA**C**GAGGTATAA**T**A**T**AGG**T**ATATAT**T**AGTATGTGGAT**T**AAAGTTT**TTT**A**T**TGGAAAAA**T**A**T**AGAA**TT**TAT**T**A**T**T**T**A**T**AA**T**AGTGA**C**G**T**A**T**ATTAA**T**T**TT**AAATTTGAATGTTTTGAATT**TT**A**T**T**TTT**ATT**T**T**T**TTATGAAAATAAATGAGGAAAA**T**TGAAGGTTAAG**T**T**T**TAT**T**AGT**T**A**T**TA**T**TTT**TT**TGAAGTAATT**T**AAAA**TT**ATT**T**TGAAT**T**TAGTGAAAGATTAAGTTGATA**T**AGAAAAGTGGGTGTG**TT**TTT**TTT**TGAGATTTGTTTTTAGATGAAGTTA**TC**GTAAGGTT**T**TTAAATTGTGTAAGT**T**TTAGTAGTTTGTGAT**T**T**T**AAA**TTC**GAAA**T**AAAAATAAAAT**TT**A**T**A**T**AAAAAG**T**AT**T**ATGTTTA**T**TGAAA**T**TAA**T**AAATATGAAGAA**T**T**TT**ATAAATTA**T**TTT**T**T**T**AAAG**T**AAAGGGTAA**T**T**T**TAT**TTT**ATTAAGGTTAAAAAAGTTAAT**T**TTTGAGT**T**AG**T**AGGTGAAAA**T**AAG**T**TGAATTATGTTGTGG**TT**TTTTT**T**TTTATTAA**T**TAAGGA**T**TTTGAA**T**TATTAAAA**T**TT**TT**TAGGGAA**TT**AGTTTT**T**T**T**AATGGGT**T**AAGT**T**A**T**A**T**AGGGGAA**T**AAAG**T**AAA**T**TTTAT**T**AGTG**T**TGTG**T**AATT**T**TA**T**ATT**TT**AGAAATT**T**TTAGTA**T**TTGG**T**ATT**T**ATATA**TT**A**T**TG**T**TGA**T**TAGAG**T**TTGAGT**T**AAT**TT**A**TT**AAA**T**T**T**AG**T**TATGG**T**TA**TT**AA**TT**TTGTGAA**T**TT**T**T**T**A**TT**AAGATA**TTT**A**T**TT**T**TTTTAAGTTAG**TT**A**C**GA**T**TAGA**T**AGG**T**AGAGTA**T**T**T**TTTAATTAAAATAGATAAATA**TT**AAGG**T**TTA**T**A**TTT**TGGGGGGA**T**ATTGTATAT**T**AGTGA**T**T**T**TTTA**T**AAAAAGAAAATATTTTTA**TT**A**TT**ATAAATTTTT**T**T**T**AAAGTGA**T**AGGT**TT** |

The qMSP primers are highlighted in yellow. The two most significant probes from the TCGA analysis (cg16506910 and cg19098437) are highlighted in green.

**Supplementary Table 4.** Cox univariable and multivariable analysis of clinicopathological and epigenetic variables in Cohort #1 (DFS).

| Disease-Free Survival | Variable | HR | 95% CI for  HR | *p* value |
| --- | --- | --- | --- | --- |
| Univariable | **miR-30a-5p expression**  ≥P50 *vs.* <P50 | 0.453 | 0.222-0.928 | 0.030 |
|  | **Clinical Stage**  I&II *vs.* III&IV | 4.339 | 2.285-8.239 | <0.0001 |
|  | **Führman Grade**  G1&G2 *vs.* G3&G4 | 2.516 | 1.197-5.288 | 0.015 |
|  | **miR-30a-5p^me^**  ≥P20 *vs.* <P20 | 3.560 | 1.096-11.564 | 0.035 |
| Multivariable | **miR-30a-5p^me^**  ≥P20 *vs.* <P20 | 3.115 | 0.957-10.142 | 0.059 |
|  | **Clinical Stage**  I&II vs. III&IV | 3.563 | 1.879-6.757 | <0.0001 |

**Supplementary Table 5.** Cox univariable analysis of clinicopathological and epigenetic variables in Cohort #1 (DSS).

| Disease-Specific Survival | Variable | HR | 95% CI for  HR | *p* value |
| --- | --- | --- | --- | --- |
| Univariable | **miR-30a-5p expression**  ≥P30 *vs.* <P30 | 0.524 | 0.283-0.970 | 0.040 |
|  | **Clinical Stage**  I&II *vs.* III&IV | 3.990 | 2.112-7.537 | <0.0001 |
|  | **Führman Grade**  G1&G2 *vs.* G3&G4 | 3.495 | 1.547-7.896 | 0.003 |
|  | **miR-30a-5p^me^**  ≥P20 *vs.* <P20 | 6.219 | 1.493-25.905 | 0.012 |

**Supplementary Table 6.** Cox univariable and multivariable analysis of clinicopathological and epigenetic variables in Cohort #2 (MFS).

| Metastasis-Free Survival | Variable | HR | 95% CI for  HR | *p* value |
| --- | --- | --- | --- | --- |
| Univariable | **miR-30a-5p^me^**  ≥P70 *vs.* <P70 | 7.436 | 1.575-35.105 | 0.011 |
|  | **Führman Grade**  G1&G2 *vs.* G3&G4 | 3.997 | 1.017-15.710 | 0.047 |
| Multivariable | **miR-30a-5p^me^**  ≥P70 *vs.* <P70 | 6.292 | 1.318-30.026 | 0.021 |
|  | **Führman Grade**  G1&G2 *vs.* G3&G4 | 3.070 | 0.778-12.114 | 0.109 |

**Supplementary Table 7.** Cox univariable analysis of clinicopathological and epigenetic variables in Cohort #2 (DSS).

| Disease-Specific Survival | Variable | HR | 95% CI for  HR | *p* value |
| --- | --- | --- | --- | --- |
| Univariable | **miR-30a-5p^me^**  ≥P70 *vs.* <P70 | 14.260 | 1.805-112.649 | 0.012 |
|  | **Führman Grade**  G1&G2 *vs.* G3&G4 | 11.705 | 1.452-94.333 | 0.021 |

**Supplementary Figure 1.** **Expression of miR-30a-5p according to clinicopathological variables in Cohort #1.** Scatter plots of miR-30a-5p expression levels according to metastasis presentation, recurrence and Führman grade (Mann–Whitney U test).

**Supplementary Figure 2. Prognostic value of stage and nuclear grade in Cohort #1.** **(A)** Disease-free and (**B**) disease-specific Kaplan-Meier survival curves based on clinicopathological stage and nuclear grade (Log-rank test).

**Supplementary Figure 3.** **MiR-30a-5p^me^ levels and prognostic value of stage and nuclear grade in Cohort #2** **(A)** Scatter plots of miR-30a-5p^me^ levels according to pathological stage (Mann–Whitney U test); **(B)** Disease-specific and **(C)** Metastasis-free Kaplan-Meier survival curves based on clinicopathological stage and nuclear grade (Log-rank test).
