# Supplementary figures and images for: Correction: MicroRNA-30a-5pme: a novel diagnostic and prognostic biomarker for clear cell renal cell carcinoma in tissue and urine samples
Source: J Exp Clin Cancer Res. 2022 Aug 15;41:247. doi: 10.1186/s13046-022-02450-x (PMC9377117; doi:10.1186/s13046-022-02450-x)

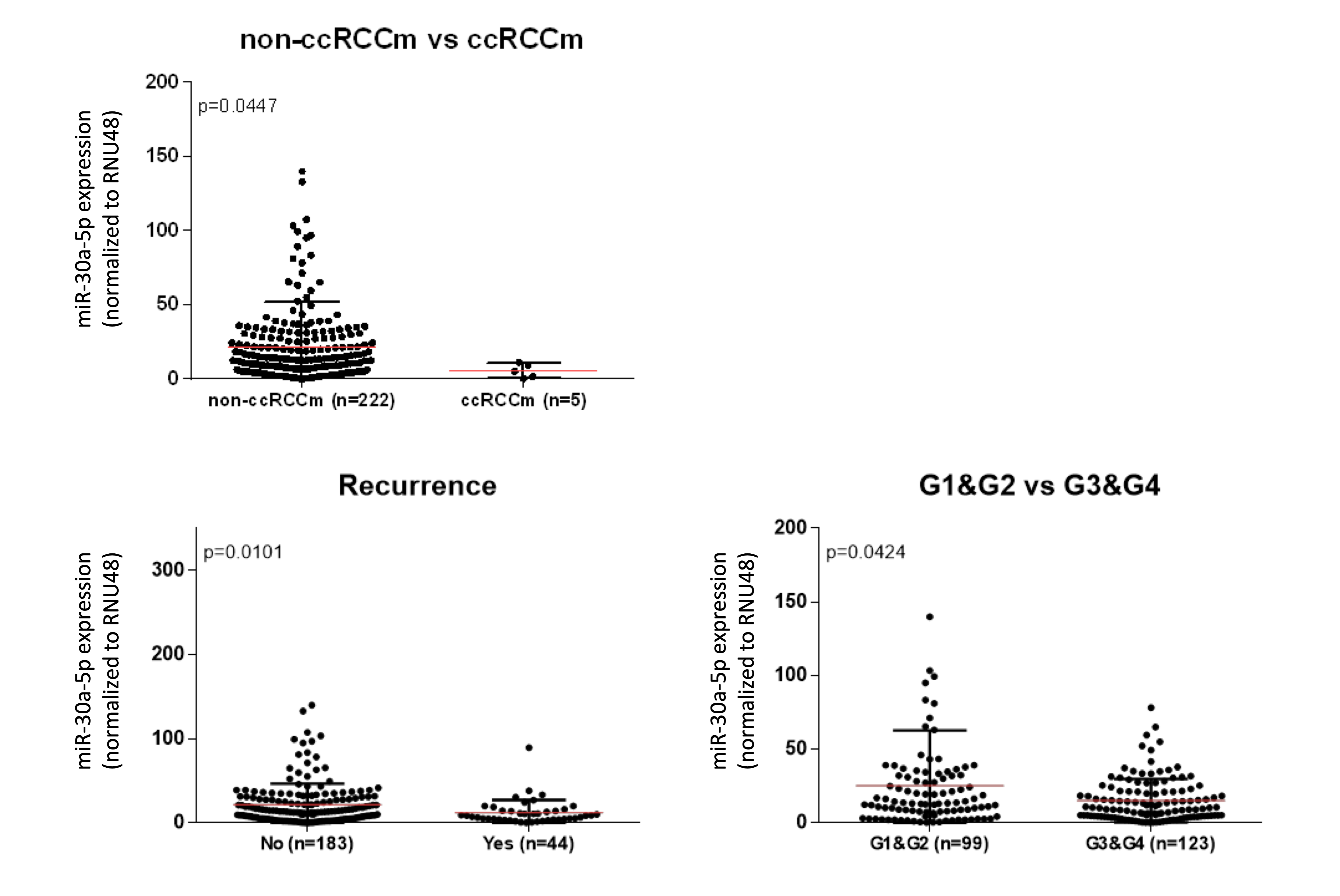

Supplement: Supplementary file 1 — Additional file 1: Supplementary Figure S1. Expression of miR-30a-5p according to clinicopathological variables in Cohort #1. Scatter plots of miR-30a-5p expression levels according to metastasis presentation, recurrence and Führman grade (Mann–Whitney U test). [file 13046_2022_2450_MOESM1_ESM.tiff]
